# The Book World of Medicine and Science

**Published:** 1901-11-16

**Authors:** 


### The Book World of Medicine and Science.

A Dictionary of Treatment or Therapeutic Index, including Medical and Surgical Therapeutics. By William Whitla, M.A., M.D. (London: Henry Renshaw. 1902. Price 16s.)

This will be found a very useful book by many practitioners. It consists of a series of articles—short essays varying from a few lines up to 20 pages in length—dealing with the treatment of a series of diseases which are arranged alphabetically. As in the old recipe, you first catch your hare, so in using this book you first find out your malady and give a name to it, when the treatment becomes literally as plain as A B C. According to the preface this work grew out of an attempt to produce a therapeutic index to Dr. Whitla's well-known book on materia medica, and it still retains traces of its origin in the number and variety of the forms of treatment suggested for some of the diseases mentioned. There are many signs, however, that in writing this dictionary the author has brought to bear upon it a large practical experience, and no one who turns up the various subjects which have lately occupied the attention of the medical world can fail to see how thoroughly the more recently-introduced methods of treatment have been incorporated in its pages. Our chief criticism would be in regard to the "dictionary" form which the work has been made to assume, and to the half-hearted manner in which the undoubted advantages of the alphabetical system have been utilised. In the first place, although it is called a "Dictionary of Treatment," there is not, so far as we can see, either a heading in the book or an entry in the index referring to modes of treatment. It is really a dictionary of diseases, giving under the head of each the treatment which may be employed. Then we cannot but feel that a dictionary which requires an index is a contradiction in terms. There are numerous cross references within the book, and the addition of a few more would have made the reader independent of the vexatious necessity of looking up his subject in the index as well as in the pages. Whenever matter is arranged alphabetically everything should appear in one list. For all that it is a useful book, and it has this very great advantage, that it covers the field. Dr. Whitla has seen, what is every year becoming more clear, that the boundary lines between medicine and surgery are purely conventional, and he has had the hardihood to throw aside the pedantic divisions of practice insisted on by some high authorities, and has included within the covers of his book the whole range of practice. Eyes, ears, noses, throats, as well as all medicine and all surgery, are included. Midwifery certainly is not to be found. Presumably, notwithstanding much that we hear nowadays, childbirth is not yet considered a disease. Still we find placenta prævia, and post-partum hæmorrhage, and adherent placenta dealt with, besides a whole crowd of gynæcological questions. So the wide scope of the work may be imagined.

Phototherapy. (1) The Chemical Rays of Light and Small-pox. (2) Light as a Stimulant. (3) The Treatment of Lupus Vulgaris by Concentrated Chemical Rays. By Professor Niels R. Finsen, Copenhagen. Translated from the German edition and with an appendix on the Light Treatment of Lupus. By James H. Sequeira, M.D.Lond., M.R.C.P. (London: Edward Arnold. 1901. Price 4s. 6d. net.)

The stream of new knowledge pours in upon us so rapidly that one soon forgets the origin of things, and no doubt by this time many have forgotten that Finsen's original research in regard to the therapeutic action of light had to do with the treatment of small-pox. For this among other reasons, as well as because at the present moment all that has to do with the treatment of small-pox has a more immediate interest than it might possess at other times, the publication of this translation of Finsen's three essays is very opportune. His original paper on the chemical rays and small-pox was published in 1894. In this he arrived at the conclusion that if the patients are subjected in time to treatment by exclusion of all but the red rays, and if the rules he lays down for the management of the cases be properly followed, suppuration will generally not occur, and the patient will recover without scars, or with only almost invisible cicatrices. An appendix, published in 1898, gives a *résumé* of the results obtained by other physicians who had made use of the treatment, results which in most cases went to confirm Professor Finsen in his conclusions. Photographs and charts are given, which fully prove the severity of some of the cases, and also the claim made for the treatment that under it the secondary fever is avoided. The portion of the book which deals with the treatment of lupus describes the now well-known details of this method, and the appendix by Dr. Sequeira gives the present position of the treatment, as shown by the results obtained abroad, as well as by those which have occurred within his own experience at the London Hospital.

The Scientific Roll and Magazine of Systematised Notes. Conducted by Alexander Ramsay. Vol I. No. 3. September, 1901. (London: R. L. Shorland. Price 1s.)

All indices are useful if they are well done, and this one seems to be carefully compiled. It is a vast undertaking to attempt, as the author here does, to catalogue and index all the literature of scientific work. So far as this periodical has gone it has dealt with the literature of bacteria, giving for each year the various works and articles which have that year appeared on the subject, with their authors' names and other details. It is an undertaking which ought to be supported by those who make use of scientific libraries.

Our Army in South Africa. By R. Scot Skirving, late Consulting Surgeon to the Australian Contingents. (London: The Australian Book Company. 1901.)

This little pamphlet is a racily-written comment on the conduct of the war, by an observer who is charged with a mighty contempt for red-tape and all its progeny. It is both entertaining and instructive, and while full of generous sympathy for the good work which was done in plenty by individuals in every branch of the Army, is a scathing sarcasm on our whole system of going to war. "It is mainly by one's mistakes that one learns. If this be so, the South African campaign must have been a marvellous school for all ranks and all departments of the Army."

